# Intermediate filaments of zebrafish retinal and optic nerve astrocytes and Müller glia: differential distribution of cytokeratin and GFAP

**DOI:** 10.1186/1756-0500-3-50

**Published:** 2010-03-01

**Authors:** Joseph R Koke, Amanda L Mosier, Dana M García

**Affiliations:** 1 Department of Biology, Texas State University-San Marcos, San Marcos, TX 78666, USA

## Abstract

**Background:**

Optic nerve regeneration (ONR) following injury is a model for central nervous system regeneration. In zebrafish, ONR is rapid - neurites cross the lesion and enter the optic tectum within 7 days; in mammals regeneration does not take place unless astrocytic reactivity is suppressed. Glial fibrillary acidic protein (GFAP) is used as a marker for retinal and optic nerve astrocytes in both fish and mammals, even though it has long been known that astrocytes of optic nerves in many fish, including zebrafish, express cytokeratins and not GFAP. We used immunofluorescence to localize GFAP and cytokeratin in wild-type zebrafish and transgenic zebrafish expressing green fluorescent protein (GFP) under control of a GFAP promoter to determine the pattern of expression of intermediate filaments in retina and optic nerve.

**Findings:**

GFAP labeling and GFAP gene expression as indicated by GFP fluorescence was found only in the Müller glial cells of the retina. Within Müller cells, GFP fluorescence filled the entire cell while GFAP labelling was more restricted in distribution. No GFAP expression was observed in optic nerves. Cytokeratin labeling of astrocytes was observed throughout the optic nerve and less intensely in cells in the retinal inner plexiform layer. The retinal inner limiting membrane was strongly labeled by anti-cytokeratin.

**Conclusions:**

Studies of astrocyte function during ONR in zebrafish cannot solely rely on GFAP as an astrocyte marker or indicator of reactivity. Future studies of ONR in zebrafish should include evaluation of changes in cytokeratin expression and localization in the optic nerve.

## Introduction

Because of the accessibility of the optic nerve, optic nerve regeneration (ONR) is often used for studies of central nervous system regeneration. In fish, typified by zebrafish, regeneration of the optic nerve after injury by crushing or transectioning is rapid with new neurites crossing the lesion and entering the optic tectum in as few as 7 days [1]. In mammals, typified by mice, regeneration does not take place in the absence of specific molecular interventions and suppression of astrocyte reactivity in the optic nerve [2,3] (for a recent review, see [4]).

As part of an ongoing study of ONR in zebrafish [5], we examined intermediate filament (IF) expression of astrocytes in the zebrafish retina and optic nerve. Many previous studies have used the type III IF glial fibrillary acidic protein (GFAP) as a marker for retinal and optic nerve astrocytes, both in fish and mammals, even though it has been known for some time that astrocytes of optic nerves in many fish, including zebrafish, express cytokeratins rather than GFAP [6,7]. A possible exception are astrocytes of goldfish optic nerve, which, as reported by Nona et al[9], appear GFAP positive both before and after optic nerve injury.

## Methods

**Figure 1:**
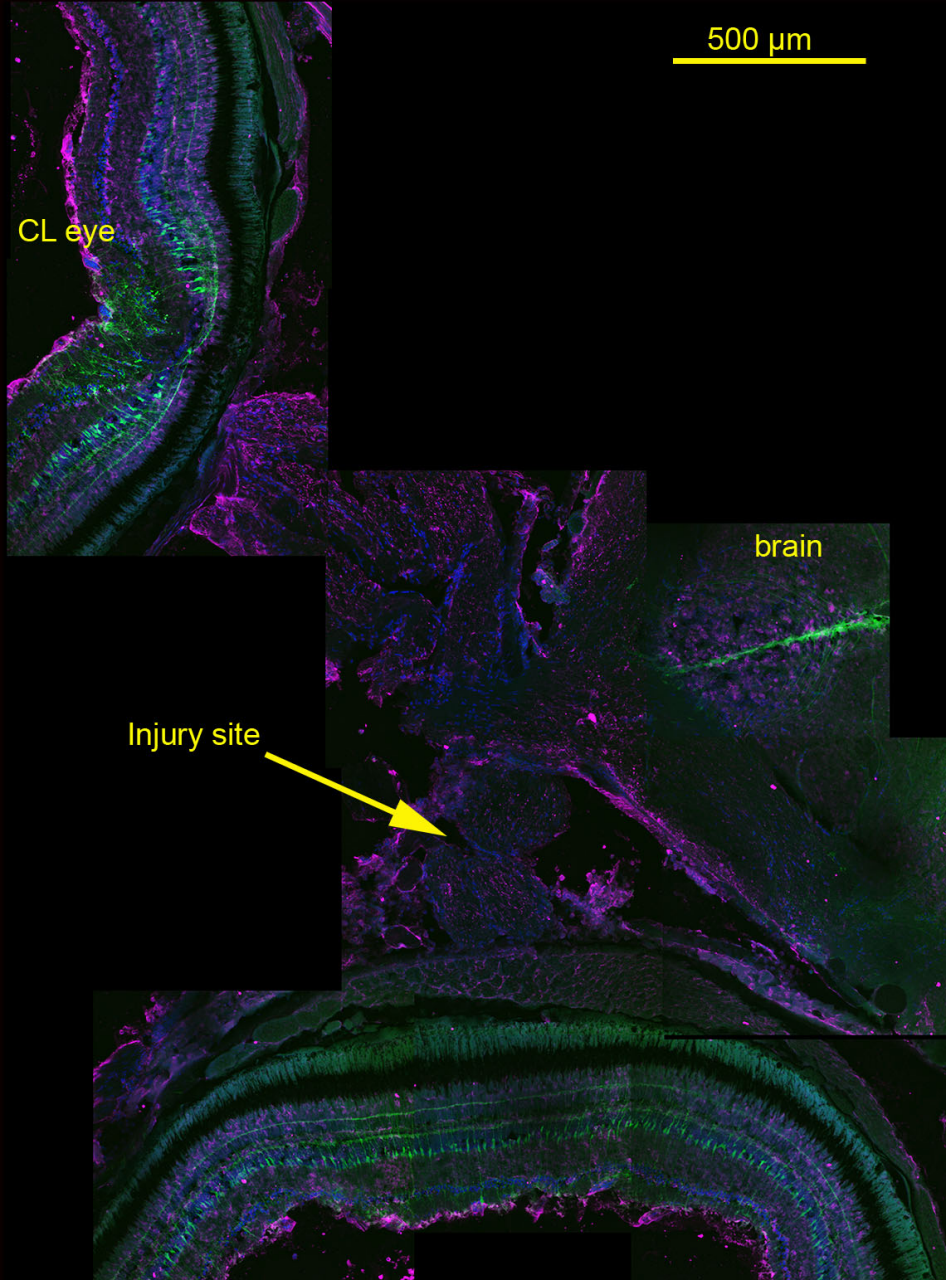
**Montage of images showing Tg(*gfap*:GFP) and cytokeratin localization in the retinas, optic nerves, and a portion of brain obtained from a fish fixed 24 hours post-optic nerve injury. (20× water immersion, NA 0.95)**. Prominent GFP expression throughout the Müller glia is visible in retinas of eye associated with the injured optic nerve (injury site, arrow) and the contralateral eye (CL eye), and delimiting what appear to be radial glia in a portion of the brain (brain). No GFP expression can be seen in the optic nerve between the retina and optic tract. Cytokeratin (magenta) labeling is apparent in the inner limiting membranes and less intensely in the cytoplasm of cells in the inner plexiform layers of the retinas and in the reticular astrocytes of the optic nerve. Blue label is DAPI indicating nuclei. See Figures 2 and 3 for enlarged views of retina and optic nerve.

All animal use protocols were approved by the Texas State University-San Marcos IACUC (approval # 0703_0122_07). Wild-type ZDR zebrafish (*Danio rerio*, Aquatica Tropicals, Plant City, FL) and transgenic zebrafish expressing green fluorescent protein (GFP) under control of a GFAP promoter were acclimated to a 12/12 hour light/dark cycle for a minimum of 14 days before use. The transgenic fish (Tg(*gfap*:GFP)^mi2001^[10]), were obtained from the Zebrafish International Resource Center, Eugene, OR. Optic nerve injury was accomplished as described in Saul et al. (2009) [5]. For immunofluorescent localization of GFAP and cytokeratin, entire fish (N = 3 each of ZDR and (Tg(*gfap*:GFP)^mi2001^) were fixed overnight in 4% formaldehyde derived by alkaline depolymerization of paraformaldehyde. Then both eyes, optic nerves and brain were dissected out intact. Following washing in PBS, the tissue was cryoprotected by incubation in 30% sucrose-PBS until the tissue sank. The intact eyes, optic nerves, chiasma, and brain were mounted to permit horizontal sectioning, allowing sections to include retinas from both the injured and contralateral sides, optic nerves, chiasma, and optic tectum of the brain. Sections were cut at 20 μm using a Zeiss Microm cryostat, collected on gelatin-coated coverslips and stored at -80°C until use. Immunostaining was performed as previously described [11], using anti-GFAP mAB 131-17719 (Molecular Probes, http://www.invitrogen.com) and anti-KRT 18 mAB (Abgent, San Diego, CA) with appropriate second antibodies conjugated respectively to TRITC and Cy5. DAPI was added to the final wash to stain nuclei. Imaging was performed using an Olympus FV1000 confocal microscopy system and sized for publication using Adobe PhotoShop CS3. Each image presented is a z-projection of 10 optical sections 1.0 μm thick for the 20× objective (NA 0.95) and 0.4 μm thick for the 60× objective (NA 1.4). The objective used is indicated in the figure legends. Figure 1 is a montage of such images.

## Results and Discussion

**Figure 2:**
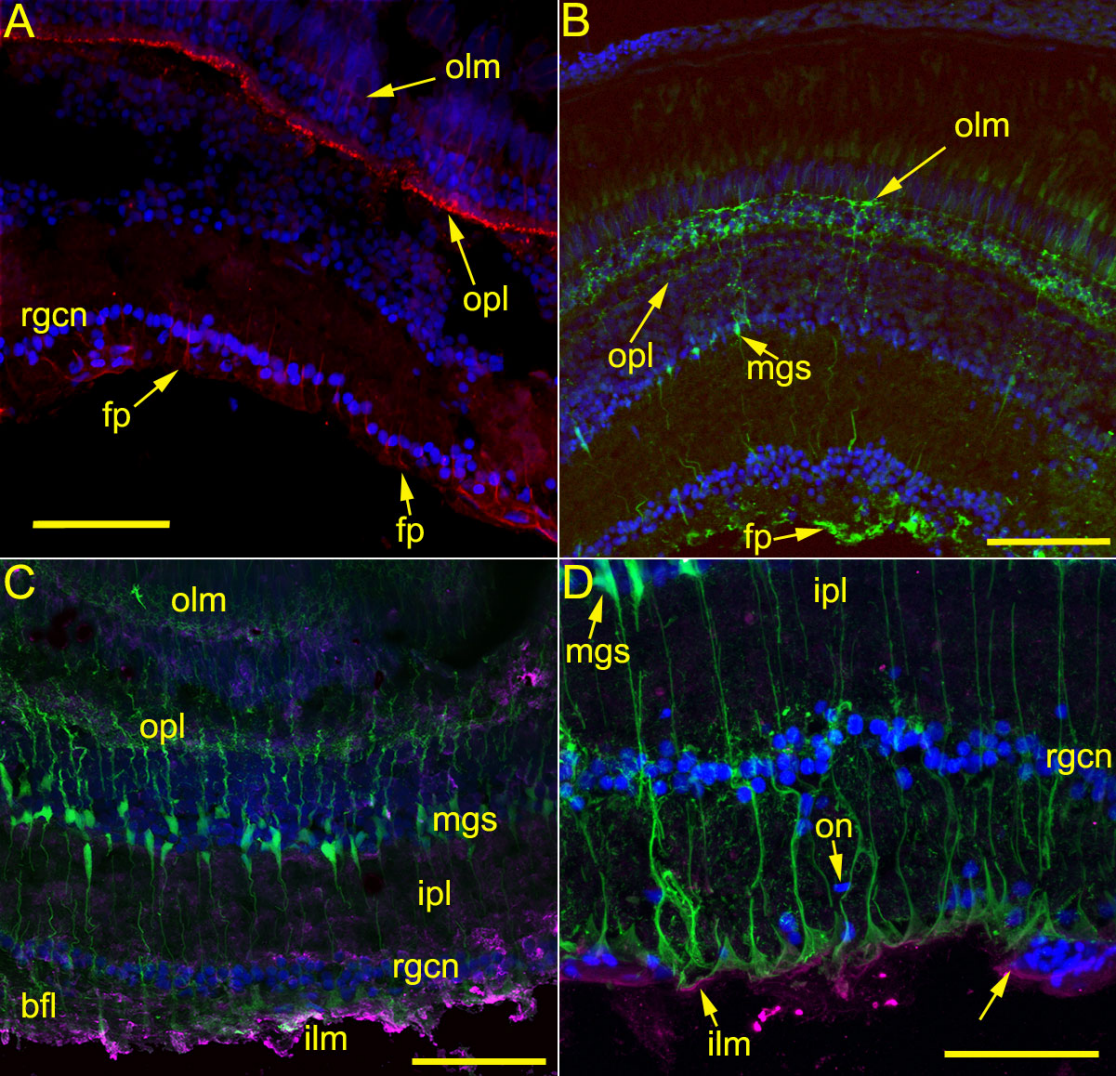
**Images of retina from ZDR (A) and Tg(*gfap*:GFP) (B, C, D) zebrafish showing localization of anti-GFAP, anti-cytokeratin, and GFP. (A, B, C, 20× water immersion, NA 0.95; D 60× oil immersion, NA 1.4)**. 2A. An image of retina from a ZDR fish immunostained with anti-GFAP (red) and DAPI (blue). Strong labeling in the foot processes (fp) of Müller glia extending between the RGC nuclei (rgcn) into the inner plexiform layer is apparent. In addition, the Müller glial elements of the outer plexiform layer (opl) are brightly decorated, with fainter labeling extending to the outer limiting membrane. The scale bar represents 55 μm. 2B. Bright GFP fluorescence can be observed in Müller glia extending from the foot processes (fp) at the inner limiting membrane, through the outer plexiform layer (opl), to the outer limiting membrane (olm). The Müller soma (mgs) are visible among the nuclei of the amacrine cells. Note the distribution of GFAP as indicated by anti-GFAP (Figure 2A) is limited to certain regions of the Müller cells and differs from distribution of GFP expressed under a transgene promoter; GFP appears to illuminate all parts of the cell. The scale bar represents 85 μm. 2C. An image of retina from Tg(*gfap*:GFP) fish has been immunostained for anti-cytokeratin (magenta). As in Figure 2B, GFP fluorescence delineates Müller glia cells from the outer limiting membrane (olm) to the inner limiting membrane (ilm). Bright anti-cytokeratin labeling can be seen on the inner limiting membrane and in the cytoplasm of cells among the bundled fiber layer (bfl). Less intense cytokeratin labeling is apparent in the inner plexiform layer (ipl). The scale bar represents 70 μm. 2D. Similar to Figure 2C, at higher magnification showing details of the inner segments of the Müller glial cells and anti-cytokeratin labeling of the inner limiting membrane (ilm). Oblong nuclei characteristic of oligodendrocytes can be seen in the nerve fiber layer. The arrow at lower right indicates a blood vessel. The scale bar represents 50 μm.

**Figure 3:**
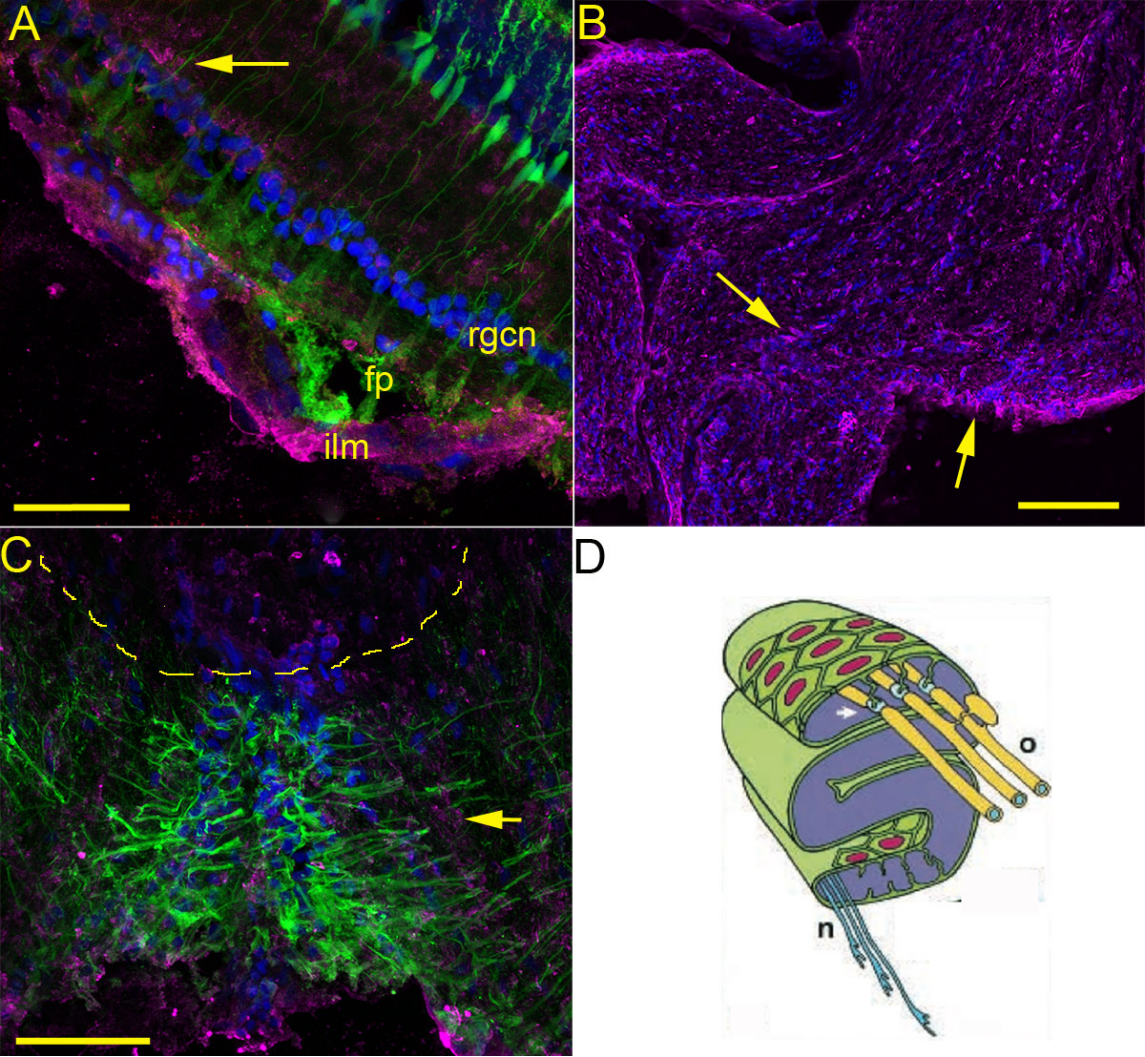
**Images of retina from Tg(gfap:GFP) (A, B, C) zebrafish showing localization of anti-cytokeratin and GFP (A, 60× oil immersion, NA 1.4; B, C, 20× water immersion, NA 0.95), and (D) a diagram showing the ribbon structure of zebrafish optic nerve**. 3A. A portion of retina labeled with anti-cytokeratin (magenta), DAPI (blue) and GFP. The inner limiting membrane (ilm) appears brightly decorated with anti-cytokeratin, which also less intensely labels cytoplasm of cells in the inner plexiform layer (example at arrow). The foot processes (fp) and inner segments of the Müller glia cells are illuminated by GFP and the nuclei of the RGCs (rgcn) by DAPI. The calibration bar represents 45 μm. 3B. Image of the optic nerve from the same section as shown in Figures 3A and 3C. Anti-cytokeratin labeling (magenta, arrows) can be seen in the cytoplasm of the optic nerve astrocytes that form the neurolemma of the ribbon-like optic nerve (see Figure 3D). Note the absence of GFP expression. The calibration bar represents 175 μm. 3C shows a section of retina that includes the optic nerve head, with the dotted line highlighting the portion of the section where the optic nerve appears exiting the retina. Cells resembling optic nerve astrocytes showing anti-cytokeratin labeled cytoplasm (magenta; example at arrow) appear to stream from the nerve into the retina. Note how the GFP-expressing Müller glial cells appear to form or strongly interact with the physiological cup of the optic nerve head, but then are excluded from the optic nerve itself. The calibration line represents 50 μm. Figure D is a diagram (adapted with permission from Figure 2D, Macdonald et al. [12]) illustrating the ribbon nature of the nerve, and the reticular astrocytes (green with red nuclei) forming the neurolemma and extending processes to the nodes (arrow, blue) in the myelin sheath formed by oligodendrocytes (O) on the RGC axons (n). The pattern of anti-cytokeratin staining seen in Figure 3B is most consistent with the Macdonald et al. [12] description of optic nerve astrocytes and cytokeratin distribution.

Sections of Tg(*gfap*:GFP) fish displayed prominent fluorescence that illuminated Müller glial cells in the retina and radial astrocytes in the brain. No GFP fluorescence was detected in the optic nerve, beginning at the optic nerve head and through the chiasma, until the optic tract (Figure 1). Anti-GFAP staining of wild-type ZDR retinae revealed strong localization of GFAP in the foot processes of Müller glia extending inward into the nerve fiber layer, and in the reticular layer formed in the synaptic zone between the photoreceptor and bipolar cells (Figure 2A). Anti-GFAP failed to label any structures in optic nerve sections (data not shown). Sections of Tg(*gfap*:GFP) retina displayed prominent GFP fluorescence in the Müller glial cells that illuminated their entire length from foot processes on the inner limiting membrane to the terminal outer limiting membrane that forms at the level of the photoreceptor ellipsoids; i.e., between the nuclei and inner segments of the photoreceptors where the intense labeling lateralizes and ends just outer to the nuclear layer (Figure 2B). GFP fluorescence also revealed fine details of the Müller cells including spines along the length of the cells and filamentous arborizations forming the foot processes (Figure 2D). Anti-cytokeratin labeling of sections of Tg(*gfap*:GFP) retina revealed antigen localization in cells making up the inner limiting membrane, in the bundled retinal ganglion cell (RGC) axon layer, and in scattered cells of the inner plexiform layer (Figure 2C, D; Figure 3A). While it is clear that in both ZDR and Tg(*gfap*:GFP) fish, Müller glia express GFAP and not cytokeratin, the differences between anti-GFAP staining in ZDR fish retina and GFP expression in the Tg(*gfap*:GFP) retina suggest that GFP localization in Tg(*gfap*:GFP) does not accurately reflect where the GFAP protein is localized in the Müller cells. This observation in turn suggests that GFAP in Müller glia contains cytoplasmic localization, or sorting, signals which GFP lacks, as suggested by Bernardos and Raymond [9], the creators of this transgenic fish.

The rapid regeneration of optic nerve in zebrafish as compared non-regeneration in mammals (who express GFAP in their optic nerves) suggests that GFAP itself is non-permissive to axonal regeneration. In mammals, reactive astrogliosis that includes upregulation of GFAP and vimentin provides a neuroprotective effect, particularly in a stroke model. Li et al. [8] found transection of the middle cerebral artery in Gfap(-/-) Vim(-/-) mice generated an infarct that was 210% to 350% larger than in wild type mice. They also report that Gfap(-/-) Vim(-/-) mice show attenuated reactive gliosis and improved post-traumatic regeneration as compared to wild type. In goldfish, Nona et al. [9] reported the presence of GFAP-positive astrocytes 7 days following an optic nerve crush injury on both proximal and distal to the lesion site; however, the injury site itself remained GFAP-negative, and astrocytes were excluded until after axonal regeneration was complete. Thus one could speculate that the absence of GFAP expression in fish optic nerve contributes to an environment that is permissive to nerve regeneration, but there seems to be no evidence that cytokeratins promote regeneration. In the present study, we found no evidence of increased cytokeratin expression in the injured optic nerve as compared to the uninjured. This result is consistent with previous studies of cytokeratin expression during optic nerve regeneration by Fuchs et al. (1994) in goldfish, where no changes in mRNA expression for the goldfish optic nerve cytokeratins GK48 and GK49 were found 10 days post-injury.

Anti-cytokeratin labeling of sections of optic nerve from Tg(*gfap*:GFP) fish revealed strong cytoplasmic labeling of cells consistent with reticular astrocytes, as described by Macdonald et al. [12] and earlier by Maggs and Scholes [13] (Figure 3B, D). As GFAP expression appears absent in zebrafish optic nerve, confirmation of these cells as astrocytes must depend on morphology, and the pattern of anti-cytokeratin staining seen in Figure 3B is most consistent with the Macdonald et al. [12] description of optic nerve astrocytes and cytokeratin distribution. Neurons would not be expected to label with anti-cytokeratin, and reports of cytokeratin expression in zebrafish oligodendrocytes are absent from the literature. Intermediate filaments of mammalian oligodendrocytes have been characterized as nestin and vimentin [14]. In contrast to the GFP expression observed in Müller glia in retina of Tg(*gfap*:GFP) fish, no GFP expression was observed in the optic nerves of the same fish. At the optic nerve head, a sharp boundary was present which appeared to exclude GFP-expressing Müller cells from the optic nerve. Cells showing cytokeratin labeling in their cytoplasm were found in the optic nerve head, and appeared to extend into the RGC nerve fiber layer, retinal inner plexiform layer, and may contribute to the inner limiting membrane (Figure 2D, 3C).

On the basis of these results, it appears that if Müller glial cells can be considered astrocytes, zebrafish have two populations of astrocytes in their retina, the GFAP expressing Müller cells, and the cytokeratin expressing reticular astrocytes that appear to extend into the retina from the optic nerve, forming the inner limiting membrane and contributing to the bundled nerve fiber and inner plexiform layers. According to Watanabe and Raff [15], a similar situation exists in mammalian retina with respect to non-Müller astrocytes entering the retina from the optic nerve along retinal vasculature, and in the mature retina, locating near the retinal vasculature and nerve fiber layer (although mammalian astrocytes do express GFAP and not cytokeratin). Because of the apparent absence of GFAP expression by any cell type in the zebrafish optic nerve - either injured or uninjured - studies of the role astrocytes may play during ONR in zebrafish cannot rely on GFAP as an marker for astrocytes or an indicator of reactivity. Future studies of ONR in zebrafish should include evaluation of changes in cytokeratin expression and localization in the optic nerve.

## Competing interests

The authors declare that they have no competing interests.

## Authors' contributions

All authors have read and approve the final manuscript. JRK contributed to the experimental design, supervised the microscopy and prepared the final images and manuscript. ALM performed the zebrafish surgeries, dissections, microtechnique and collected the images. DMG conceived and supervised the overall project and provided intellectual guidance.
